# Behavioral interventions to promote adequate sleep among women: protocol for a systematic review and meta-analysis

**DOI:** 10.1186/s13643-017-0490-y

**Published:** 2017-05-11

**Authors:** Lydi-Anne Vézina-Im, Jennette Palcic Moreno, Theresa A. Nicklas, Tom Baranowski

**Affiliations:** Children’s Nutrition Research Center, Baylor College of Medicine, 1100 Bates Street, Houston, TX USA 77030

**Keywords:** Sleep, Intervention, Women, Behavior, Systematic review, Protocol

## Abstract

**Background:**

Short and poor sleep have been associated with adverse health outcomes in adults, such as overweight/obesity and type 2 diabetes, especially among women. Women therefore represent an important target for interventions aimed at improving sleep and such interventions have been advocated to enhance maternal, fetal, and infant health. This systematic review will assess the efficacy or effectiveness of behavioral interventions aimed at promoting adequate sleep among women. The primary outcomes will be changes in sleep duration and/or sleep quality from baseline to post-intervention and to the last available follow-up measured either through self-reports or objectively. Secondary outcomes will be assessing the behavior change techniques that are responsible for the changes in sleep duration and quality among women.

**Methods:**

Behavioral interventions that are non-pharmacological and target either sleep directly or sleep hygiene behaviors will be included. Randomized controlled trials, quasi-experimental, and one-group pre-post studies will be included, but treated separately in the analyses, given that a limited number of studies on the topic of sleep is expected. MEDLINE/PubMed, PsycINFO, CINAHL, EMBASE, and Proquest Dissertations and Theses will be investigated. There will be no restriction on the year of publication of the articles, but we will include only the ones written in English or French. Two authors will independently assess articles for eligibility and will extract data using a standardized data extraction form that will have been previously pilot-tested. The quality of the studies will be assessed using the Effective Public Health Practice Project tool for quantitative study designs. The intervention procedures will be classified according to the latest validated taxonomy of behavior change techniques. If there is a sufficient number of studies (*k* > 5), a meta-analysis of the results will be performed with a random-effect model. If the heterogeneity is high (*I* ^2^ ≥ 75%), it will be investigated through sensitivity analyses and meta-regression.

**Discussion:**

This review will provide valuable information to those interested in promoting adequate sleep among women and, hopefully, encourage additional research in this important field to promote maternal, fetal, and infant health.

**Systematic review registration:**

The study protocol was registered in PROSPERO in October 2016 (CRD42016049538).

**Electronic supplementary material:**

The online version of this article (doi:10.1186/s13643-017-0490-y) contains supplementary material, which is available to authorized users.

## Background

Recently, many studies have found inverse associations between sleep and both high adiposity [1–6] and type 2 diabetes [7] in adults. Two aspects of sleep were related to adverse health outcomes: duration and quality. Most of these studies focused on sleep duration. In the United States of America, the National Sleep Foundation recommends between 7 and 9 h of sleep per night for young adults and adults [8]. However, definitions of short sleep duration vary from one study to another. Among adults, short sleep duration has been associated with overweight and obesity [6, 9–13], either measured in terms of body mass index (BMI) [1, 3] or waist circumference [1, 5], and with impaired fasting glucose [14, 15], prediabetes [16], and type 2 diabetes [7, 13, 15, 17].

Other adverse outcomes associated with short sleep duration are earlier mortality, hypertension, cardiovascular, and coronary heart diseases [13]. The association between mortality and sleep duration occurred when people slept less than 6 h at night [13]. Short sleep duration has also been linked to obesity in women [18–22]. Maternal short sleep duration (≤5 h per day) in the first year postpartum was associated with higher adiposity at 3 years postpartum [23], shorter sleep duration during pregnancy was linked to increased risk for gestational hyperglycemia [24] and very short sleep (≤4 h per night) with gestational diabetes mellitus [25].

A few studies have assessed sleep quality (i.e., number of awakenings at night or the subjective perception of how one’s sleep is restorative [26]) and its impact on health. Similar to short sleep duration, poor sleep quality was associated with overweight/obesity [27, 28], risk of diabetes [29], prediabetes [16], and type 2 diabetes [30–32] in adults. Women seemed more at risk for poor sleep [33] and insomnia [34] compared to men, and poor sleep was linked with high adiposity in women [19, 28]. Poor sleep quality was also associated with impaired fasting glucose in women with previous gestational diabetes mellitus [35]. In the USA, women between the ages of 15 and 44 years (i.e., of childbearing age) were especially at risk for poor sleep when compared with pregnant women of the same age range [36].

In sum, short and poor sleep are important risk factors for obesity and type 2 diabetes among adults, especially among women [18, 20, 21]. Women represent an important target for interventions aimed at improving sleep given that they are especially at risk for short [36] and poor sleep [33]. Moreover, when women are of childbearing age, an intervention promoting sleep can impact both the woman’s and the child’s health by addressing prenatal predictors of obesity, such as pre-pregnancy BMI [37]. Pre-pregnancy BMI can also be an independent risk factor for gestational diabetes mellitus [38]. Therefore, interventions to promote adequate sleep have been advocated to enhance maternal, fetal, and infant health [39].

Previous reviews of behavioral or non-pharmacological sleep interventions have either focused on underage populations, such as infants [40, 41] or children [42–44]; older people [45, 46]; populations with health problems, such as youth with chronic health conditions [47], children with autism spectrum disorders [48], hospitalized patients [49], oncology patients and family caregivers [50], adults in intensive care units [51]; or people with sleep disorders, such as apnea [52–54] or insomnia [55, 56]; or on specific interventions, such as stress reduction [57].

These reviews included the following types of behavioral sleep interventions: educational interventions (e.g., providing parents information on safe sleep among infants [40] or on positive routines to favor sleep in their child [42, 44]), sleep education [43, 50] or sleep hygiene education [46, 49], cognitive behavioral therapy [43, 50, 55, 56] or cognitive therapy [46], environmental interventions (e.g., reducing environmental noise in aged care facilities [45]), relaxation [49, 51, 55, 56] or massage [51] or aromatherapy [51] or stress reduction [57], and exercise [50] or lifestyle, and dietary interventions [52, 53].

Some reviews on behavioral sleep interventions conducted among adults have found somewhat conflicting results. For example, one review on the effects of mindfulness-based stress reduction conducted among adults—mostly female patients with chronic diseases, such as cancer and fibromyalgia—found that this technique was associated with statistically significant improvements in sleep quality or duration in studies with a pre-post design [57]. However, this was no longer the case in randomized controlled trials; there were no significant differences in sleep between the experimental and the control groups [57]. Some reviews also reported that the use of relaxation techniques—something akin to mindfulness-based stress reduction techniques—can improve sleep quality in hospitalized patients [49] and in patients in intensive care units [51]. It is therefore difficult to determine if behavioral interventions, such as relaxation or stress reduction techniques, are effective at improving sleep duration and/or quality; which types of behavioral interventions are the most effective at promoting sleep; and if the results of reviews conducted in adults apply specifically to women.

A search in MEDLINE/PubMed, the Cochrane Library, and PROSPERO on September 27, 2016 confirmed that there was no published systematic review/meta-analysis or published protocol for a systematic review on behavioral interventions to promote adequate sleep among women. This systematic review will fill this gap in the scientific literature by assessing the efficacy or effectiveness of behavioral interventions aimed at promoting adequate sleep among women. The review will answer the following questions:

- What is the impact of behavioral interventions promoting sleep among women on sleep duration and/or sleep quality?
- Which components of the interventions are effective at changing sleep duration and/or sleep quality?
- What biases are present in the peer-reviewed published articles?
- Do the effects of the interventions vary by the quality of the study or characteristics of the participants?

## Methods

The study protocol was registered in PROSPERO (www.crd.york.ac.uk/PROSPERO/) in October 2016 (CRD42016049538). The outline of this protocol follows the Preferred Reporting Items for Systematic Review and Meta-analysis Protocols (PRISMA-P) checklist [58]. Additional file 1 shows the completed PRISMA-P checklist.

### Study eligibility criteria

#### Population

Only studies reporting sleep data on adult women (over 18 years of age) will be included. Studies on special populations, such as women with health problems (e.g., breast cancer) or sleep disorders (e.g., apnea) will also be included. If a study reports sleep data on men and women separately, only the data on women will be included and analyzed. In instances where the sleep data in adults is not separated by gender, the authors of the articles will be personally contacted to verify if they can provide the data pertaining only to women.

#### Intervention

Behavioral interventions that are non-pharmacological (i.e., no use of sleep medication) and target either sleep directly or sleep hygiene behaviors, such as caffeine consumption, will be included. Some behavioral interventions might target multiple behaviors, such as sleep, physical activity, and diet. In those instances, only the information on sleep will be included, but the inclusiveness of the intervention targets will be recorded.

#### Outcome

The primary outcomes will be changes in sleep duration (in minutes or hours) from baseline (i.e., pre-intervention) to post-intervention and to the last available follow-up, measured either through self-reports or objectively (e.g., by wrist actigraphy) and changes in sleep quality from baseline (i.e., pre-intervention) to post-intervention and to the last available follow-up measured either through self-reports (e.g., Pittsburgh Sleep Quality Index [59]) or objectively (e.g., number of awakenings at night measured by wrist actigraphy). The secondary outcomes will be assessing the components of the behavioral interventions, such as behavior change techniques, that are responsible for the changes in sleep duration and quality among women.

#### Study designs

Randomized controlled trials (RCT), quasi-experimental studies, and one-group pre-post studies will be included, but treated separately in the analyses. One-group pre-post studies will be included, since sleep is a rather new target for behavior change and this will allow the inclusion of pilot studies. Also, a preliminary search of the scientific literature indicated that there are few interventions targeting sleep compared to other health-related behaviors, such as physical activity and diet. For studies with a control group, the following types of control will be included: no-treatment, wait list, usual care, and alternative treatment.

### Search strategy

The following databases will be investigated: MEDLINE/PubMed (1950+), PsycINFO (1806+), CINAHL (1982+), and EMBASE (1974+). Proquest Dissertations and Theses (1861+) will also be investigated for unpublished trials (a form of grey literature). There will be no restriction on the year nor the country of publication of the articles. In each database, the search strategy will include terms related to three major themes: sleep, women, and interventions. Additional file 2 presents the complete search strategy for MEDLINE/PubMed. Additional studies will be included by checking the references of the articles included in the systematic review (i.e., secondary references). Only studies written in English and French will be included. The results of the search strategy will be reported in a Preferred Reporting Items for Systematic Reviews and Meta-analyses (PRISMA) flow-chart [60].

### Study selection and data extraction

All the articles retrieved from the search strategy will first be screened for possible duplicates. Next, clearly irrelevant articles will be excluded according to their title and abstract. The remaining articles will be fully retrieved (full-text), and two authors will independently assess them for eligibility. We will also verify if some studies report results based on the same sample and/or the same intervention. In those instances, we will only select the study with the best methodological qualities (e.g., RCT versus pilot study) and that report the most information (e.g., baseline, post-intervention, and follow-up data versus baseline and post-intervention data only) to avoid duplication of results and attributing more weight to these studies in the meta-analysis of the results.

Data will be extracted independently by two reviewers using a standardized data extraction form that will previously be pilot-tested with five randomly selected articles. The quality of the studies will be assessed using the Quality Assessment Tool for Quantitative Study of the Effective Public Health Practice Project (EPHPP) [61]. This tool is recommended by the Cochrane Collaboration [62] and can be used for different kinds of quantitative study designs (e.g., RCT, quasi-experimental studies, and one-group pre-post studies). Briefly, the tool evaluates the quality of studies using the following six criteria: (1) selection bias (2), study design (3), confounders (4), blinding (5), data collection method, and (6) withdrawals and dropouts. The rating for each of the six components is used to obtain a global rating of a study’s quality. A strong rating is obtained when there are no weak ratings to any of the six components of the EPHPP tool. A moderate global rating is obtained when there is one weak rating and a weak global rating when there are two or more (out of 6) weak ratings [61]. The components of the intervention will be classified according to a validated taxonomy of behavior change techniques [63] which contains 93 different behavior change techniques. Disagreements at each step will be resolved by discussion. When no consensus is reached, a third reviewer will resolve the discrepancy.

### Data synthesis and analyses

The results of the included studies will be reported descriptively in a summary table which will present information on the objective of the intervention, the population targeted, the study design, and the quality of the study according to the EPHPP tool, the baseline characteristics of the sample, the type of intervention and the use of theory, the behavior change techniques used, the behavioral measure, and the results on sleep duration and/or quality. The results on sleep duration and/or quality will be reported in effect sizes, such as odds ratios (OR) for dichotomous outcomes (e.g., improvement in sleep quality: yes/no) or the standardized mean differences (SMD) for continuous outcomes (e.g., increase in hours in sleep duration). All effect sizes will be zero order (i.e., no covariates will be included in the computation of the effect size). The OR will be converted to Cohen’s d [64] to facilitate interpretation and to allow comparison with other SMD and standard effect sizes reported in other studies. A Cohen’s d of 0.20 is considered a small effect size, 0.50 a medium effect size, and 0.80 a large effect size [64].

If there is a sufficient number of studies reporting similar interventions among a similar population (*k* > 5) [65], a meta-analysis of the results will be performed with a random-effect model. A random-effect will be used for all the analyses because we expect that the magnitude of the effect sizes will vary across studies given the differences in samples and interventions across studies [66]. Each study will be weighted according to its sample size when we compute the pooled effect sizes. Between-study heterogeneity will be verified using a Cochran’s *Q* [67] and the *I* ^2^ statistic [68] as measures of the percentage of total variation in estimated effects that is due to heterogeneity rather than chance [69]. A significant *Q* statistic (*p* < 0.05) indicates significant heterogeneity between the studies while an I^2^ squared statistic of 25% is considered low heterogeneity, 50% moderate heterogeneity, and 75% high heterogeneity [70]. If between-study heterogeneity is high (I^2^ ≥ 75%), it will be investigated through sensitivity analyses and meta-regression. Publication bias will be assessed by visually inspecting the distribution of the funnel plot when there will be at least ten studies per analysis [71], by using Duval and Tweedie’s [72] “trim and fill” method, and by using Egger’s [73] regression test. Comprehensive Meta-Analysis software, version 3 [74] will be used to conduct all the analyses.

If the necessary data are available, subgroup analyses will be done to verify the efficacy or effectiveness of interventions promoting sleep according to the following variables: population (e.g., healthy women versus women with medical conditions), participant characteristics (e.g., age, socioeconomic status), study design, quality rating according to the EPHPP tool, type of intervention, use of theory (e.g., theory-based versus non-theory-based interventions), use of a formal planning process (e.g., Intervention Mapping [75], Behavior Change Wheel [76]), and behavior change techniques used.

## Discussion

This review will provide important information on the efficacy or effectiveness of behavioral interventions to promote adequate sleep duration and quality among women. By investigating both sleep duration and sleep quality, the review will allow researchers, clinicians and public health stakeholders to know if the majority of sleep interventions targeted sleep duration, sleep quality, or both, and whether their efficacy or effectiveness varied according to which aspect of sleep was targeted. It will also provide valuable information by identifying which components—or behavior change techniques—were effective at changing women’s sleep duration and/or quality. Identifying which techniques were more effective or more promising at changing sleep could encourage further research on the use of these strategies to promote adequate sleep among women, an effective way to accumulate scientific evidence and advance knowledge on behavior change. In sum, this review will identify gaps in knowledge and will provide useful recommendations for those interested in promoting adequate sleep among women. It is also hoped that it will encourage additional research on how to improve sleep among women, since they represent an important target for sleep interventions and such interventions have the unique potential to promote both maternal, fetal and infant health [39].

## Additional files

Additional file 1: PRISMA-P + checklist.docx: Completed PRISMA-P checklist. (DOCX 29 kb)

Additional file 2: Search Strategy for PubMed.docx: complete search strategy for MEDLINE/PubMed. (DOCX 15 kb)

## Abbreviations

BMI: Body mass index
EPHPP: Effective Public Health Practice Project
OR: Odds ratio
PRISMA: Preferred reporting items for systematic reviews and meta-analyses
PRISMA-P: Preferred reporting items for systematic review and meta-analysis protocols
RCT: Randomized controlled trial
SMD: Standardized mean difference
