# Supplementary material for: Behavioral interventions to promote adequate sleep among women: protocol for a systematic review and meta-analysis
Source: Syst Rev. 2017 May 11;6:95. doi: 10.1186/s13643-017-0490-y (PMC5425988; doi:10.1186/s13643-017-0490-y)
Supplement: Supplementary file 2 — Search Strategy for PubMed.docx: complete search strategy for MEDLINE/PubMed. (DOCX 15 kb) [file 13643_2017_490_MOESM2_ESM.docx]

Additional File 2. Search Strategy

| Name of the database (range of dates) | MEDLINE/PubMed (1950+) |
| --- | --- |
| Dates of the search |  |
| Initials of the person who will run the search | LAVI |
| Search terms / MeSH | (“sleep”[Title]) OR (“insomnia”[Title]) OR (“sleep disorder*”[Title]) OR (“sleep”[MeSH Major Topic]) or (“sleep initiation and maintenance disorders”[MeSH Major Topic]” OR (“sleep wake disorders, circadian rhythm”[MeSH Major Topic])  AND  (“women”[MeSH Major Topic]) OR (“pregnant women”[MeSH Major Topic])  AND  (“intervention*”[Title]) OR (“education*”[Title]” OR (“program*”[Title]) OR (“treatment*”[Title]) OR (“behavio* therapy”[Title]) OR (“health promotion”[MeSH Major Topic]) |
| Limits | -Article types: clinical trial, controlled clinical trial, randomized controlled trial  -Species: humans  -Languages: English, French  -Sex: female |
| Number of hits |  |
